# Association of frailty with clinical and financial outcomes of esophagectomy hospitalizations in the United States^☆^

**DOI:** 10.1016/j.sopen.2022.05.003

**Published:** 2022-05-19

**Authors:** Mina G Park, Greg Haro, Russyan Mark Mabeza, Sara Sakowitz, Arjun Verma, Cory Lee, Catherine Williamson, Peyman Benharash

**Affiliations:** a Cardiovascular Outcomes Research Laboratories, Division of Cardiac Surgery, David Geffen School of Medicine at UCLA, Los Angeles, CA; b Department of Surgery, David Geffen School of Medicine at UCLA, Los Angeles, CA, USA

## Abstract

**Background:**

*Frailty*, defined as impaired physiologic reserve and function, has been associated with inferior results after surgery. Using a coding-based tool, we examined the clinical and financial impact of frailty on outcomes following esophagectomy.

**Methods:**

Adults undergoing elective esophagectomy were identified using the 2010–2018 Nationwide Readmissions Database. Using the binary Johns Hopkins Adjusted Clinical Groups frailty indicator, patients were classified as frail or nonfrail. Multivariable regression models were used to evaluate the association of frailty with in-hospital mortality, complications, hospitalization duration, costs, nonhome discharge, and unplanned 30-day readmission.

**Results:**

Of 45,361 patients who underwent esophagectomy, 18.7% were considered frail. Most frail patients were found to have diagnoses of malnutrition (70%) or weight loss (15%) at the time of surgery. After adjustment, frailty was associated with increased risk of in-hospital mortality (adjusted odds ratio 1.67, 95% confidence interval 1.29–2.16) and overall complications (adjusted odds ratio 1.57, 95% confidence interval 1.44–1.71). Frailty conferred a 5.6-day increment in length of stay (95% confidence interval 4.94–6.45) and an additional $19,900 hospitalization cost (95% confidence interval $16,700–$23,100). Frail patients had increased odds of nonhome discharge (adjusted odds ratio 1.53, 95% confidence interval 1.35–1.75) as well as unplanned 30-day readmissions (adjusted odds ratio 1.17, 95% confidence interval 1.02–1.34).

**Conclusion:**

Frailty, as detected by an administrative tool, is associated with worse clinical and financial outcomes following esophagectomy. The inclusion of standardized assessment of frailty in risk models may better inform patient selection and shared decision-making prior to operative intervention.

## INTRODUCTION

Despite significant advances in surgical technique and care, esophagectomy remains a high-risk operation with mortality rates up to 10% and postoperative complications occurring in nearly 40% of patients [1,2]. Careful risk assessment is crucial to patient selection and shared decision-making when choosing treatment options for esophageal disease. Although a multitude of traditional models incorporate patient age and comorbidities to estimate perioperative risk, the concept of frailty has emerged as an important predictor of adverse events [[1], [2], [3]]. Mounting evidence has linked frailty with increased mortality, complications, and health care expenditures following major operations including coronary artery bypass grafting, lung resection, and colectomy [[4], [5], [6]].

Although the exact definition of frailty remains equivocal, several physical and cognitive tests have been developed to characterize its presence. However, such frailty assessment methods have not been widely adopted in surgical practice because of their resource-intensive nature [7]. More recently, algorithms relying on administrate data have been employed to readily identify frailty. In fact, the American College of Surgeons National Surgical Quality Improvement Project (NSQIP) has incorporated several iterations of a coding-based frailty index into their risk models [8]. The addition of frailty has generally improved the discriminatory power of prediction models to better identify at-risk subject [8]. In an NSQIP study of 2095 esophagectomy patients, Hodari et al found a 30-fold increase in postoperative mortality in the presence of frailty [2].

With the known limitations of the Modified Frailty Index and the NSQIP database including selective center participation, the Johns Hopkins Adjusted Clinical Groups (ACG) frailty indicator has been introduced [[9], [10], [11]]. The ACG relies on diagnosis codes and can thus be widely applied to administrative data. Our group and several other investigators have previously reported on the significant impact of ACG classification on postoperative outcomes [[4], [5], [6]]. However, the utility of this frailty indicator in predicting risk of esophagectomy at the national level has not been evaluated.

The present national study characterized the association of the frailty, as measured by the ACG, with clinical outcomes and resource use following esophagectomy. We hypothesized frailty to be independently associated with increased risk of in-hospital mortality, perioperative complications, length of stay, hospitalization costs, and 30-day readmissions.

## METHODS

The 2010–2018 Nationwide Readmissions Database (NRD) was queried to identify all elective, adult (≥ 18 years) hospitalizations for esophagectomy. The NRD is a national, all-payer database maintained by the Healthcare Cost and Utilization Project (HCUP) that accrues data from 27 states and provides accurate estimates for approximately 60% of all US hospitalizations. Previously reported *International Classification of Diseases, Ninth and Tenth Edition, Clinical Modification* (*ICD-9*/*10-CM*) diagnosis and procedure codes were used to identify hospitalizations for esophagectomy for benign and malignant indications [12]. Records with missing data for age, sex, in-hospital mortality, or hospitalization costs were excluded (4.1%).

Frailty was defined according to the Johns Hopkins Adjusted Clinical Groups diagnosis clusters, which included malnutrition, dementia, severe visual impairment, decubitus pressure ulcer, urinary incontinence, fecal incontinence, poverty, difficulty walking, and falls. Specific *ICD* diagnosis codes used to identify frailty qualifying diagnoses can be found in Supplementary Table 1. Patients were categorized as FRAIL if any 1 of the aforementioned diagnoses were present, and all others comprised the nonfrail cohort (nFRAIL).

Patient and hospital characteristics of interest included age, sex, insurance payer, as well as hospital teaching status and bed size as defined in the HCUP data dictionary (NRD). The van Walraven modification of the Elixhauser Comorbidity Index, a previously validated composite score of 30 comorbidities, was used to quantify the burden of chronic conditions [13]. Patient comorbidities including history of chemoradiation therapy and complications of interest were also identified using relevant *ICD-9*/*10* diagnosis codes. Perioperative complications were grouped as cerebrovascular, cardiac, respiratory, gastrointestinal, infectious, and thrombotic as previously described [6,14]. Specifically, *gastrointestinal complication* was defined as a composite of bowel ischemia, intestinal abscess, intestinal fistula, atraumatic intestinal perforation, megacolon, hemoperitoneum, and postprocedural intestinal dysfunction. Anastomotic leak was not included because the correlating ICD codes were inconsistent with previously reported values (< 1%) [12,15]. Hospitalization costs were calculated using center-specific cost-to-charge ratios provided by HCUP and adjusted for inflation using the 2018 Personal Health Care Index [16]. The primary outcome of interest was in-hospital mortality, whereas secondary end points included perioperative complications, length of stay (LOS), index hospitalization costs, nonhome discharge, and 30-day unplanned readmissions.

Cuzick's nonparametric test (NPtrend) was used to assess the significance of temporal trends [17]. Categorical and continuous variables are reported as proportions or mean with standard deviation (SD) or median with interquartile range [IQR] if not normally distributed, respectively. Bivariate comparisons for categorical variables were performed using Pearson *χ*^2^ test, whereas the adjusted Wald or Mann–Whitney *U* tests were used for continuous variables. Multivariable logistic and linear regression models were developed to evaluate the independent association of frailty with outcomes of interest. Model covariates were chosen using elastic net regularization, which uses a regressive least squares methodology to minimize collinearity and applies penalties to prevent overfitting [18]. The area under the receiver-operating characteristic as well as the Akaike's and Bayesian Information Criteria was used to optimize models, as appropriate. Regression outcomes are reported as adjusted odds ratios (AORs) and *β* coefficients with 95% confidence intervals (CIs). All statistical analyses were performed using Stata 16.1 (StataCorp, College Station, TX). This study was deemed exempt from full review by the University of California, Los Angeles Institutional Review Board.

## RESULTS

**Fig 1:**
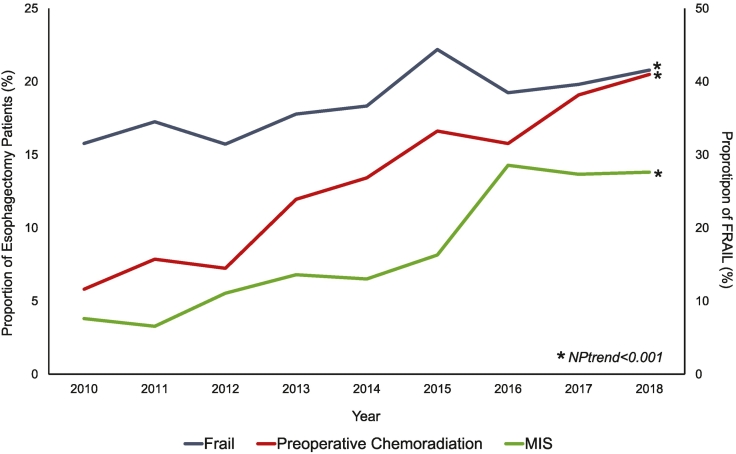
Annual proportion of frail patients undergoing elective esophagectomy stratified by minimally invasive approach and history of preoperative chemoradiation. *MIS*, minimally invasive surgery.

Of an estimated 45,361 esophagectomy hospitalizations included for analysis, 8,490 (18.7%) comprised the FRAIL cohort. Malnutrition (70.0%) followed by weight loss (14.8%), dementia (6.9%), and pressure ulcers (6.4%) were among the most common frailty qualifying diagnoses (Table 1). Within the study period, the incidence of frailty increased from 16% in 2010 to 21% in 2018 (NPtrend < .001). The rates of preoperative chemoradiation and minimally invasive surgery increased among the FRAIL patients over 9 years (Fig 1).

**Table 1:** Prevalence of Johns Hopkins ACG frailty defining diagnosis clusters within the FRAIL cohort

*ACG cluster*	*Representative diagnoses*	*Prevalence (%)*
Malnutrition	Nutritional marasmus Severe protein-calorie malnutrition	70.0
Weight loss	Abnormal weight loss Adult failure to thrive	14.8
Dementia	Presenile dementia Senile dementia Alzheimer dementia Frontotemporal dementia Unspecified dementia	6.9
Severe vision impairment	Legal blindness Blindness in both eyes	0.4
Decubitus ulcer	Decubitus ulcer	6.4
Urinary incontinence	Atony of bladder Incontinence without sensory awareness Continuous leakage Mixed incontinence Other functional disorders of bladder	0.1
Fecal incontinence	Fecal incontinence	0.3
Social needs support	Inadequate housing Confined mobility	0.2
Difficulty in walking	Abnormalities in gait and walking Difficulty walking	0.8
Falls	Falls on and from stairs and steps Falls on same level	0.1

Compared to others, patients in the FRAIL cohort were older (64.5 ± 11 vs 63.4 ± 10.9 years, *P* < .001) and had a higher Elixhauser Comorbidity Index (4 [3–5] vs 3 [2–4]), *P* < .001). Specifically, congestive heart disease, coagulopathy, and liver disease were more common among the FRAIL cohort (Table 2). FRAIL patients had a higher prevalence of esophageal cancer (53.5% vs 49.2%, *P* < .001), esophageal stricture (7.0% vs 4.2%, *P* < .001), and achalasia (4.0% vs 2.7%, *P* < .001) but lower rates of Barrett esophagus (6.1% vs 10.9%, *P* < .001) compared to others. Furthermore, FRAIL patients more frequently underwent open operations (81.7% vs 78.9%, *P* = .009) at large institutions (81.2% vs 77.1%, *P* = .006) and were insured by Medicare (51.5% vs 46.8%, *P* < .001).

**Table 2:** Demographics and clinical characteristics of patients undergoing esophagectomy stratified by frailty

	*FRAIL*	*nFRAIL*	
	*(*n *= 8,490)*	*(*n *= 36,872)*	P *value*
Age (y, SD)	64.5 ± 11.0	63.4 ± 10.9	<.001
Female (%)	23.2	23.9	.427
Elixhauser Comorbidity Index [IQR]	4 [3–5]	3 [2–4]	<.001
Indication for surgery (%)			
Malignancy	80.4	78.8	.101
History of chemoradiation	28.2	28.9	.51
Comorbidities (%)			
Congestive heart failure	6.3	4.7	<.001
Coronary artery disease	12.2	15.3	<.001
Diabetes	15.7	18.5	.001
Hypothyroidism	6.7	8.4	.003
Chronic liver disease	6.0	4.8	.008
Coagulopathy	8.5	5.4	<.001
Anemia	2.8	2.3	.16
Insurance coverage (%)			<.001
Private	35.2	42.7	
Medicare	51.5	46.8	
Medicaid	8.8	6.9	
Other payer⁎	4.5	3.6	
Operative approach (%)			.020
Open	81.7	78.9	
Laparoscopic	11.4	13.4	
Robotic	6.9	7.7	
Hospital teaching status (%)			.001
Nonmetropolitan	1.8	1.0	
Metropolitan nonteaching	8.4	9.1	
Metropolitan teaching	89.8	89.9	

Continuous variables are reported as mean with standard deviation or median with IQR.

⁎ Indicates a combined insurance status including self-pay, uninsured, and other.

On unadjusted analysis, the rates of in-hospital mortality (6.1% vs 2.9%, *P* < .001) and perioperative complications such as respiratory (37.6% vs 23.3%, *P* < .001), infectious (19.5% vs 9.5%, *P* < .001), and gastrointestinal (14.1% vs 8.5%, *P* < .001) were higher in the FRAIL cohort when compared to their counterparts (Table 3). Furthermore, FRAIL patients experienced longer LOS (13 [9–23] vs 9 [7–14] days, *P* < .001) and incurred greater hospitalization costs ($53,800 [$35,700–$89,500] vs $39,400 [$26,800–$59,600], *P* < .001). Rates of nonhome discharge (23.9% vs 11.9%, *P* < .001) and 30-day unplanned readmission (16.3% vs 13.1%, *P* < .001) were also higher in the FRAIL.

**Table 3:** Unadjusted outcomes following elective esophagectomy stratified by frailty.

	*FRAIL (*n *= 8,490)*	*nFRAIL (*n *= 36,872)*	P *value*
In-hospital mortality	6.1	2.9	<.001
Complications			
Cardiac	4.9	2.9	<.001
Respiratory	37.6	23.3	<.001
Gastroenterological	14.1	8.5	<.001
Infectious	19.9	9.5	<.001
Cerebrovascular	0.6	0.3	.032
Venous thromboembolic	3.6	2.1	<.001
Nonhome discharge	23.9	11.9	<.001
30-d nonelective readmission	16.3	13.1	<.001
LOS (d) [IQR]	13 [9–23]	9 [7–14]	<.001
Costs ($1,000) [IQR]	51.3 [34.3–87.8]	37.9 [26.9–56.6]	<.001

All outcomes reported as percentage for dichotomous variables and median with IQR for continuous variables.

**Fig 2:**
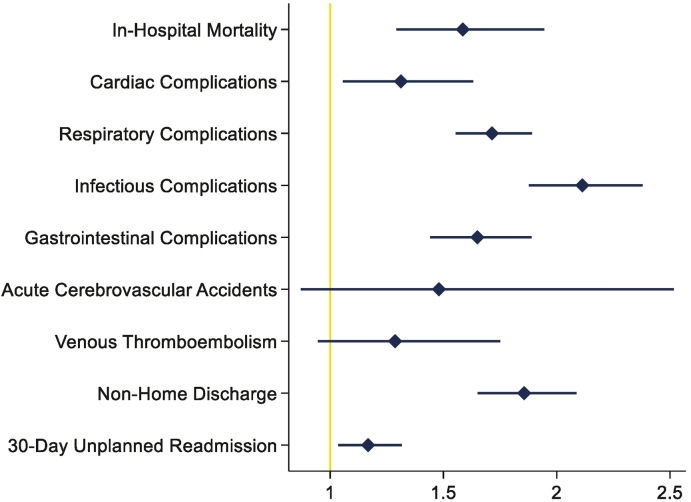
Association of frailty with mortality and perioperative complications following esophagectomy.

After multivariable risk adjustment, frailty remained independently associated with increased odds of in-hospital mortality (AOR 1.59, 95% CI 1.29–1.95, Table 4). Frailty was further linked with a greater likelihood of developing respiratory, gastrointestinal, and infectious complications (Fig 2). Moreover, frailty conferred a 5.6-day incremental increase in LOS (95% CI 4.8–6.4) and +$19,900 in hospitalization costs (95% CI $16,700–$23,100). Frailty was associated with 53% and 17% increase in relative odds of nonhome discharge (95% CI 1.35–1.75) and 30-day unplanned readmission (95% CI 1.02–1.34, Fig 2), respectively.

**Table 4:** Risk-adjusted multivariable regression model for in-hospital mortality following elective esophagectomy.

	*AOR (95% CI)*	P *value*
Year (per year)	0.96 (0.92–0.99)	.021
Patient demographics		
Age (per year)	1.04 (1.03–1.06)	<.001
Female	0.98 (0.77–1.24)	.85
Frailty	1.59 (1.29–1.95)	<.001
Elixhauser Comorbidity Index	1.12 (1.05–1.19)	<.001
Indication		
Malignant	1.27 (0.92–1.75)	.15
Benign	0.61 (0.50–0.75)	<.001
History of chemoradiation	0.52 (0.41–0.66)	<.001
Comorbidities		
Congestive heart failure	2.07 (1.54–2.77)	<.001
Coronary artery disease	0.65 (0.49–0.86)	.002
Diabetes	0.72 (0.54–0.96)	.023
Hypothyroidism	0.31 (0.20–0.49)	<.001
Liver disease	2.39 (1.78–3.23)	<.001
Coagulopathy	2.02 (1.52–2.68)	<.001
Anemia	0.54 (0.21–1.38)	.20
Payer type		
Private	Ref	
Medicare	1.44 (1.10–1.88)	.006
Medicaid	1.57 (1.04–2.36)	.030
Other⁎	2.21 (1.37–3.58)	.00
Operative characteristics		
Open	Ref	
Laparoscopic	0.56 (0.41–0.75)	<.001
Robotic	0.58 (0.42–0.82)	.002
Hospital teaching status		
Rural	Ref	
Urban nonteaching	1.08 (0.45–2.6)	.87
Urban teaching	0.74 (0.32–1.74)	.50

*Ref*, reference.

⁎ Indicates a combined insurance status including self-pay, uninsured, and other.

**Fig 3:**
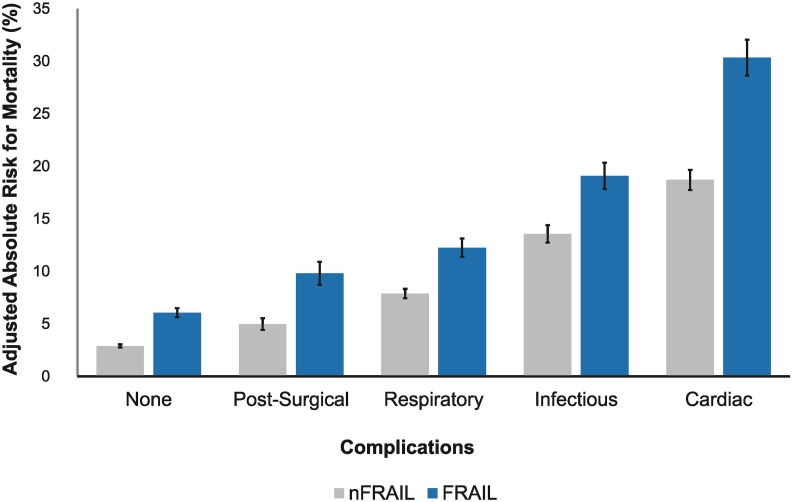
Adjusted absolute risk of in-hospital mortality associated with various complications in frail and nonfrail patients.

Risk-adjusted estimates for in-hospital mortality by frailty status was calculated using the outputs of various multivariable logistic regressions. Frailty incurred a greater increment in adjusted mortality in the presence of several complications (Fig 3). Among all complication types, cardiac complication (30.3%, 95% CI 19.8–44.2 vs 18.7% 95% CI 12.2–27.3) was associated with the largest absolute difference in death between FRAIL and nFRAIL cohorts.

## DISCUSSION

In the present nationally representative study, we examined the association of frailty, as measured by a coding-based method, with postoperative outcomes following elective esophagectomy and made several important observations. Using the proprietary ACG frailty tool, nearly 1 in 5 patients undergoing esophagectomy was classified as frail. We observed frailty to be independently associated with increased in-hospital mortality and postoperative complications. Additionally, frailty was linked with greater duration of hospitalization, costs, and risk of 30-day unplanned readmissions. Importantly, the present work demonstrates the feasibility of an administrative frailty instrument in refining risk prediction models for those undergoing elective esophagectomy.

Over the last decade, the concept of frailty has expanded beyond the normal aging process and accumulation of comorbidities [19]. Although more than 30 frailty tools have been reported, none have been adopted as standardized method of assessing frailty [7]. A simplified coding-based screening tool, like ACG frailty indicator, can be readily implemented into existing electronic medical record systems to enable timely screening, referrals, and appropriate specific evaluations of at-risk individuals. A myriad of previous works has studied the discriminatory power of ACG model of frailty in identifying frailty in the general elderly population as well as its association with poor perioperative outcomes across various surgical cohorts [4,6,20,21]. Because esophageal disease requires complex coordination of care across multiple disciplines, an administrative frailty screening tool may provide great benefit in streamlining referrals for ancillary services such as physical therapy, nutritional health, and wound care depending on the specific frailty qualifying diagnoses of the patient. For example, a simple automated alert system in the electronic medical records may provide a more realistic discussion of perioperative risk while allowing for implantation of targeted strategies for optimization. Unlike other administrative tools, ACG indicator does not include common comorbidities that overlap with traditional surgical risks but integrates specific domains of functional dependencies identifying specific areas for intervention. A broad application of ICD-based frailty indicator tool may allow for automated incorporation into risk models and facilitate choice of therapy as well as shared decision-making.

Using the Johns Hopkins ACG frailty indicator, we found nearly 20% of esophagectomy patients to be frail and > 70% having a diagnosis of malnutrition. Hodari and colleagues observed similar rates of frailty (24%) using the modified Frailty Index in the National Surgical Quality Improvement Program database [2]. However, prealbumin, a marker of nutritional status, was not found to be associated with in-hospital mortality in their multivariable regression analysis [2]. Malnutrition and weight loss are well-known risk factors for esophagectomy patients and have independently been shown to portend poor outcomes across surgical specialties [22,23]. Although laboratory values are not available in the NRD, we noted frailty to be associated with 67% increase in the relative risk of mortality and complications particularly respiratory, infectious, and gastrointestinal. This finding is congruent with prior studies of patients undergoing esophagectomy and cardiac and lung operations [2,5,23]. Although frailty itself cannot be completely reversed, preoperative nutritional evaluation and optimization may improve outcomes for frail patients undergoing esophagectomy [22,23]. Furthermore, identifying the interaction between frailty, malnutrition, and high rates of respiratory and infectious complications in esophagectomy patients suggests early postoperative integration of ancillary services such as respiratory therapy and wound care. Cao and colleagues conducted a meta-analysis examining the effects of preoperative nutritional optimization for esophagectomy candidates and found a 50% reduction in infectious complications and a 2-day decrement in LOS [24]. Implementation of the ACG frailty tool in clinical settings may provide timely prompts to intervene and optimize esophagectomy candidates in a more standardized manner.

Our work highlights the significant burden of frailty on expenditures following elective esophagectomy. Following adjustment for other risk factors, patients classified as frail experienced an additional 4 days in length of stay and incurred an excess of nearly $15,000 in index hospitalization costs. These findings may be attributable to the presence of postoperative complications as well as intensity of care, a variable that could not be measured in NRD. Our results corroborate prior work noting a similar association in coronary artery bypass surgery and lung resections [4,5]. Among those surviving index hospitalizations, 24% of frail patients required postoperative rehabilitation facilities upon discharge compared to 12% in the nonfrail cohort. Moreover, frail patients were 17% more likely to be readmitted within 30 days of discharge. Taken together, our findings point to major clinical and financial implications of frailty in surgical practice, which may be mitigated with early detection and optimization. Patient selection and shared decision-making of different treatment modalities, such as definitive chemoradiation, may be better guided with a calibrated prediction of clinical and financial risk.

The present study has several important limitations. As an administrative database, the NRD is influenced by local coding practices. Furthermore, the database does not account for outpatient care, and our analysis is limited to index hospitalizations and readmissions. Granular clinical data, such as tumor location and staging, method of esophagectomy, and the use of neoadjuvant chemotherapy or radiation, were not captured. Despite these limitations, we used the largest available all-payer readmissions database and robust statistical methods to reduce the risk of bias.

In conclusion, we found frailty, as measured by an administrative tool, to be independently associated with increased in-hospital mortality, postoperative complications, and resource utilization among patients undergoing esophagectomy. Implementation of the ACG frailty indicator into routine clinical evaluation may aid risk stratification, shared decision-making, and optimization of esophagectomy candidates.

The following are the supplementary data related to this article.

Supplementary Table 1 *ICD-9*/*10* diagnosis codes for Johns Hopkins ACG frailty qualifying categories Supplementary Table 1

## Author Contribution

All authors have contributed to the manuscript.

## Conflict of Interests

The authors have no related conflicts of interest to declare.

## Funding Source

None.

## Ethics Approval

This study was deemed exempt from full review by Internal Review Board at University of California Los Angeles.
