# Supplementary material for: Association of frailty with clinical and financial outcomes of esophagectomy hospitalizations in the United States
Source: Surg Open Sci. 2022 May 19;9:80–5. doi: 10.1016/j.sopen.2022.05.003 (PMC9198451; doi:10.1016/j.sopen.2022.05.003)
Supplement: Supplementary Table 1 — ICD-9/10 diagnosis codes for Johns Hopkins ACG frailty qualifying categories [file mmc1.docx]

**Supplementary Table 1.** International Classification of Disease (ICD) 9/10 diagnosis codes for Johns Hopkins ACG frailty qualifying categories.

| **Category** | **Representative Diagnoses** | **ICD-9/10 Codes** |
| --- | --- | --- |
| Malnutrition | Nutritional Marasmus Severe Protein-Calorie Malnutrition | 261, 262, 263.8, 263.9, V77.2, E41, E43, E44, E45, E46 |
| Dementia | Presenile Dementia, Senile Dementia  Alzheimer’s Disease  Frontotemporal Dementia  Unspecified Dementia | 290.1, 290.2, 290.3, 290.4, F01, F02, F03, F05, G30, G31.0 |
| Severe Vision Impairment | Legal Blindness  Blindness, Both Eyes | 369.0, 369.00, 369.01, 369.03, 369.04, 369.06, 369.07, 369.08, H54.0X, H54.1, H54.8 |
| Decubitus Ulcer | Decubitus Ulcer | 707.0, 707.00, 707.01, 707.02, 707.03, 707.04, 707.05, 707.06, 707.07, 707.09, 707.20, 707.21, 707.22, 707.23, 707.24, 707.25, L89 |
| Urinary Incontinence | Atony of bladder  Incontinence without Sensory Awareness Continuous Leakage Mixed Incontinence  Other Functional Disorders of Bladder | 596.4, 596.5, 788.34, 788.37, N31, N36.4, N39.42, N39.45, N39.46 |
| Fecal Incontinence | Fecal Incontinence | 787.6, R15 |
| Weight Loss | Abnormal Weight Loss Adult Failure to Thrive | 783.2, 783.21, 783.22, 783.3, R62.7, R63.0, R63.3, R63.4 |
| Social Needs Support | Inadequate Housing  Confined Mobility | V60.0, V60.1, V60.2 Z59.0, Z59.1, Z59.4, Z59.7, Z59.8, Z59.9, Z74, Z75.0, Z75.1, Z75.3, Z75.4, Y93E, Y93.F, Y93.G |
| Difficulty in Walking | Abnormalities in Gait and Walking  Difficulty in Walking | 719.7, 781.2, R26, R27, Z99.3 |
| Falls | Falls on and from Stairs and Steps Fall on Same Level | E880, E880.0, E880.1, E880.9, E884.3, W00.0XXA, W00.1XXA, W00.2XXA, W00.9XXA, W01.0XXA, W01.10XA, W01.110A, W01.111A, W01.118A, W03.XXXA, W04.XXXA, W05.0XXA, W05.2XXA, W06.XXXA, W07.XXXA, W08.XXXA, W10.0XXA, W10.1XXA, W10.2XXA, W10.8XXA, W10.9XXA, W17.81XA, W17.89XA, W18.00XA, W18.01XA, W18.02XA, W18.09XA, W18.11XA, W18.12XA, W18.31XA, W18.39XA, W18.40XA, W18.41XA, W18.42XA, W18.49XA, W19.XXXA |

ICD: International Classification of Diseases; ACG: Adjusted Clinical Group
